# Enhancing photo-catalytic production of organic acids in the cyanobacterium *Synechocystis* sp. PCC 6803 Δ*glgC*, a strain incapable of glycogen storage

**DOI:** 10.1111/1751-7915.12243

**Published:** 2015-01-23

**Authors:** Damian Carrieri, Charlie Broadbent, David Carruth, Troy Paddock, Justin Ungerer, Pin-Ching Maness, Maria Ghirardi, Jianping Yu

**Affiliations:** Biosciences Center, National Renewable Energy Laboratory15013 Denver West Parkway, Golden, CO, 80401, USA

## Abstract

A key objective in microbial biofuels strain development is to maximize carbon flux to target products while minimizing cell biomass accumulation, such that ideally the algae and bacteria would operate in a photo-catalytic state. A brief period of such a physiological state has recently been demonstrated in the cyanobacterium *Synechocystis sp.* PCC 6803 Δ*glgC* strain incapable of glycogen storage. When deprived of nitrogen, the Δ*glgC* excretes the organic acids alpha-ketoglutarate and pyruvate for a number of days without increasing cell biomass. This study examines the relationship between the growth state and the photo-catalytic state, and characterizes the metabolic adaptability of the photo-catalytic state to increasing light intensity. It is found that the culture can transition naturally from the growth state into the photo-catalytic state when provided with limited nitrogen supply during the growth phase. Photosynthetic capacity and pigments are lost over time in the photo-catalytic state. Reversal to growth state is observed with re-addition of nitrogen nutrient, accompanied by restoration of photosynthetic capacity and pigment levels in the cells. While the overall productivity increased under high light conditions, the ratio of alpha-ketoglutarate/pyruvate is altered, suggesting that carbon partition between the two products is adaptable to environmental conditions.

## Introduction

Algae including cyanobacteria are receiving increasing attention for their potential in direct photosynthetic conversion of carbon dioxide into fuels and chemicals. Compared with land plants, algae can achieve higher areal productivity and do not have to use arable land and fresh water, thus do not compete with food production for these valuable natural resources (Dismukes *et al*., 2008). To realize the potential of photosynthetic microorganisms in renewable chemical production, a strategy to minimize growth while maximizing photosynthetic productivity of target chemicals is ideal (Melis, 2012). In such an approach, a culture would not grow and instead dedicate fixed carbon from photosynthesis to production of target compounds. Towards that vision, we have genetically modified the model cyanobacterium *Synechocystis sp.* PCC 6803 (hereafter referred to as S6803) to make it incapable of glycogen storage by deleting the gene at locus slr1176 (*glgC*), which encodes a glucose-1-phosphate adenylyltransferase. When deprived of nitrogen, the wild-type (WT) strain increases cell biomass with glycogen accumulation, while the mutant instead produces and excretes alpha-ketoglutarate and pyruvate without biomass increase. The mutant continues to photosynthesize at rates similar to WT (although both WT and mutant strains decline in photosynthetic rates after nitrogen removal) and utilize newly fixed atmospheric carbon, instead of stored carbon, to produce the excreted keto acids (Carrieri *et al*., 2012). Similar findings have also been reported by other researchers and with other strains (Gründel *et al*., 2012; Hickman *et al*., 2013). In *Synechococcus elongatus* PCC 7942 and *Synechococcus sp.* PCC 7002, the *glgC* deletion mutant has been used to improve yields of targeted products with expression of heterologous pathways (Davies *et al*., 2014; Li *et al*., 2012). Excretion of keto acids by a glycogen synthesis mutant was also reported to occur under mixotrophic conditions with glucose supplementation and is considered a manifestation of overflow metabolism (Gründel *et al*., 2012). However, the photo-catalytic conversion process declines over several days and remains to be optimized for enhanced productivity.

This study aims to understand the relationship between the growth state and the photo-catalytic state observed in nitrogen starvation conditions. The approach is to identify physiological parameters involved in the transition into and out of photo-catalysis as well as the adaptability of cells in photo-catalysis to changes in environmental conditions such as increasing light intensity.

## Results and discussion

Our initial communication reporting the ability of S6803 Δ*glgC* to produce extracellular organic acids noted a severe decline in whole-chain photosynthetic capacity, a decline that was identical in rate and magnitude to that of WT. In S6803 WT, the decreased photosynthetic rates correlated with a diminishing rate of glycogen storage, while in the Δ*glgC*, concentrations of exogenous organic acids pyruvate and alpha-ketoglutarate did not increase after 3 days of continuous photo-catalysis (Carrieri *et al*., 2012). A strong decline in photosynthetic capacity under nitrogen starvation is well documented in cyanobacteria and results in dramatic transcriptional and physiological responses, which affect, among other pathways, photosynthesis and carbon fixation (Sauer *et al*., 2001; Krasikov *et al*., 2012). However, we had found that during photoautotrophic growth in the presence of nitrate, S6803 Δ*glgC* did not produce detectable amounts of extracellular organic acids. Because NO_3_^−^ is the sole nitrogen source in standard BG11 medium, we expected that nitrogen availability was critical to production of organic acids.

**Figure 1:**
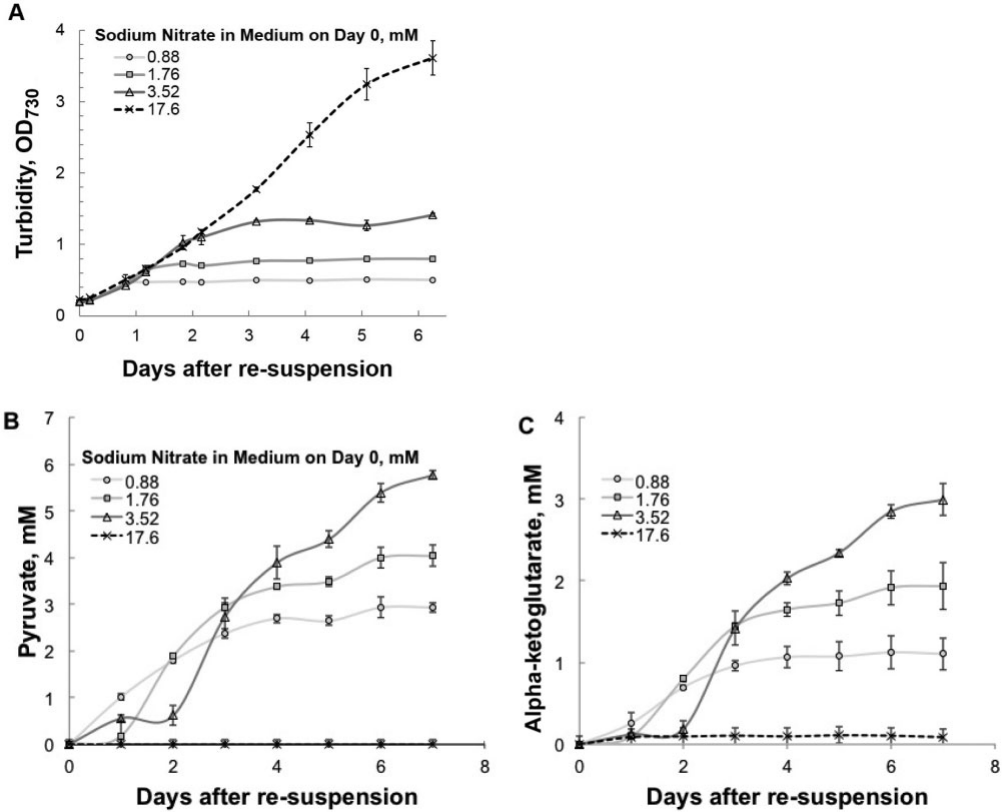
Accumulated concentration of extracellular pyruvate and alpha-ketoglutarate in batch-grown cultures of S6803 Δ*glgC* with limited nitrate in the inoculum. Cells were grown to an OD_730_ of approximately 1.0 in BG-11 (containing 17.6 mM NaNO_3_) under 50 μE* m^−2^ s^−1^ illumination from cool white fluorescent bulbs. These precultures were inoculated into BG-11 media with the indicated nitrate content and cultivated under the same illumination. Cultures were sampled regularly to measure (A) cell density (as OD_730_), (B) extracellular pyruvate and (C) extracellular alpha-ketoglutarate.

We reasoned that by providing a limiting concentration of nitrate in growth medium, cells of S6803 Δ*glgC* might assimilate all available nitrogen and transition without further manipulation into the photo-catalytic production of pyruvate and alpha-ketoglutarate. We found that while cultures in standard BG-11, which contains 17.6 mM NaNO_3_, grow to cell densities beyond 3.0 OD_730_ without excreting organic acids, growth of cells cultivated in modified medium containing 5–20% of this original nitrate concentration was arrested in a nitrate concentration dependent manner, coincident with the start of pyruvate and alpha-ketoglutarate production (Fig. 1).

This finding shows that S6803 Δ*glgC* can naturally transition from growth state to photo-catalytic state and offers a solution to an otherwise insurmountable problem for large-scale production of organic acids from this strain by nitrogen removal. Harvesting and washing, followed by re-suspension of cells in nitrate-free medium at a production-scale, would likely be prohibitively costly and energy intensive.

**Figure 2:**
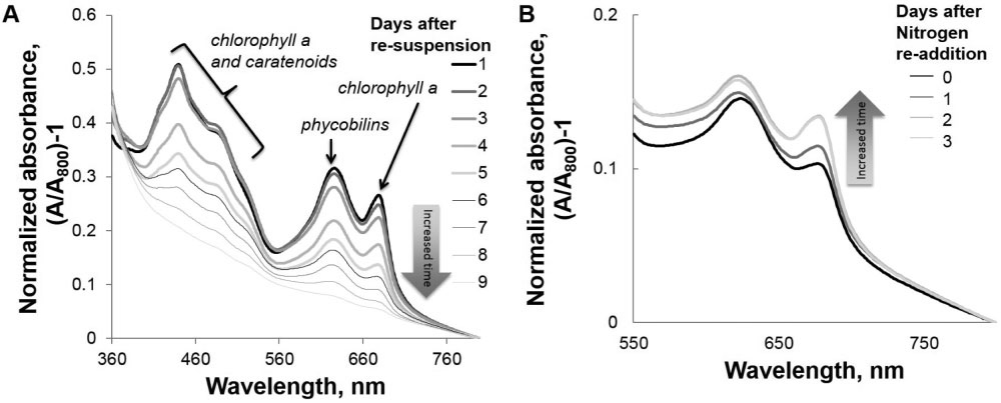
Whole-cell spectra of nitrate-free cultures of S6803 Δ*glgC* (A) over 9 days of photo-catalysis and (B) upon re-addition of 2 mM NaNO_3_ following 6 days of photo-catalysis. Cultures grown for 6 days in nitrate-limited medium in A are the same as those in B, labelled 0 days after nitrogen re-addition. Illumination was supplied by cool white fluorescent bulbs at an intensity of 50 μE* m^−2^ s^−1^.

Nevertheless, the approach taken in Fig. 1 does not address the problem of cellular photo-catalyst degradation over time. We observed degradation of pigments in whole cells of S6803 Δ*glgC* over time by taking whole-cell absorption spectra daily during the photo-catalysis phase. Figure 2A shows that after prolonged and continuous photo-catalysis (approximately 3 days after nitrogen removal in this experiment), cultures begin to lose all pigments involved in photosynthesis. This general decline in carotenoids, chlorophyll a, and bilins within phycobilisome is different from the rapid degradation of pigments associated with nitrogen limited cyanobacterial wild-types. In wild-type cyanobacteria, nitrogen deprivation results in degradation of phycobilisome within hours and a much slower decline in chlorophyll a (Sauer *et al*., 2001; Carrieri *et al*., 2012; Krasikov *et al*., 2012). It is not yet understood why *glgC* deletion mutants do not rapidly degrade their phycobilisomes under nitrogen-depleted conditions.

Nevertheless, similar to what is reported for wild-type cells (e.g. Krasikov *et al*., 2012), we found that the level of pigments in nitrogen-free cultures of S6803 Δ*glgC* can increase again upon nitrate re-addition. Even after 6 days of illumination of nitrate-free cultures of S6803 Δ*glgC*, pigments increased in cultures upon addition of 2 mM NaNO_3_ (Fig. 2B). This suggested that perhaps the photo-catalyst could be regenerated in S6803 Δ*glgC*.

**Figure 3:**
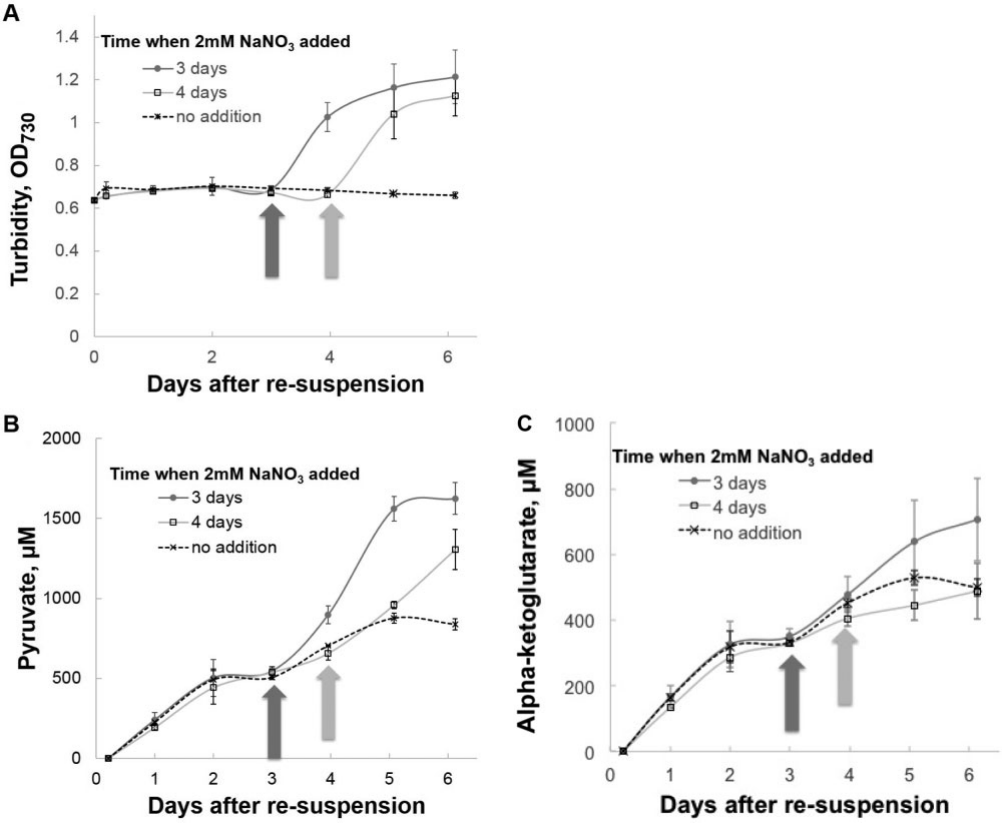
Regeneration of photo-catalytic production of pyruvate and alpha-ketoglutarate from nitrate-free cell suspensions of S6803 Δ*glgC* by addition of 2 mM NaNO_3_ after 3 or 4 days of photo-catalysis. Illumination was supplied by cool white fluorescent bulbs at an intensity of 50 μE* m^−2^ s^−1^. Cells were grown to an OD_730_ of approximately 1.0 in BG-11 (containing 17.6 mM NaNO_3_) under 50 μE* m^−2^ s^−1^ from cool white fluorescent bulbs and re-suspended after washing in nitrate-free BG-11 to an OD_730_ of approximately 0.6. Cultures were sampled regularly to measure (A) cell density (as OD_730_), (B) extracellular pyruvate and (C) extracellular alpha-ketoglutarate.

We therefore reverted to our original conditions for production of organic acids by S6803 Δ*glgC* and compared them with cultures in which 2 mM NaNO_3_ was added after three or four days of photo-catalysis, as shown in Fig. 3. As expected, re-addition of nitrate allowed for cell growth to resume (as indicated by turbidity measurements in Fig. 3A), followed by production of more organic acids pyruvate (3B) and alpha-ketoglutarate (3C) than in control cultures.

These data suggest that the photo-catalytic state is reversible at the culture level and that photobioreactors producing pyruvate and alpha-ketoglutarate with S6803 Δ*glgC* could likely be regenerated for at least two cycles of photo-catalysis. We should note, however, that, based on our data, we cannot distinguish whether the newly generated cells after nitrate re-addition were responsible for recovery of pigments and photo-catalytic activity of cultures or individual cells recovered from inactivity to production. The latter explanation is plausible, considering that in the cyanobacterium *S. elongatus* PCC 7942, long-term nitrogen deprivation of WT cells leads to a survivable but low-level photosynthetic state (Sauer *et al*., 2001). Regeneration of the whole-cell photo-catalyst could inspire further research on this physiological state and further optimization towards development of a high carbon efficiency process for photosynthetic production of fuels and chemicals.

**Figure 4:**
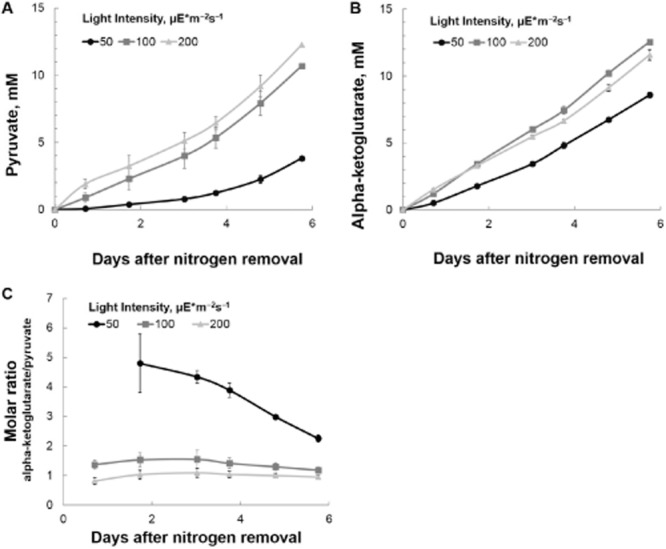
Effect of continuous illumination intensity on photo-catalytic production of (A) pyruvate and (B) alpha-ketoglutarate, and (C) the calculated molar ratio of alpha-ketoglutarate/pyruvate yield. Cultures of S6803 Δ*glgC* were grown under 24 h of illumination from cool white LEDs at the indicated light intensities, harvested and washed in nitrate-free BG-11, and re-suspended at a cell density of 3.1 OD_730_. The new suspensions were returned to the same conditions in which they were grown and sampled daily to measure extracellular metabolites. The molar ratio of products from cultures illuminated at 50 μE* m^−2^s^−1^ had high uncertainty (because of detection limits of high-performance liquid chromatography for pyruvate) prior to 1 day after nitrogen removal and is therefore not shown in panel C.

Finally, we supposed that under certain conditions, light should be limiting for photo-catalytic production of organic acids and that higher light flux should result in faster generation of products. This result has been observed in many other cyanobacterial systems (e.g. Halfmann *et al*., 2014). To test this hypothesis, we cultured S6803 Δ*glgC* cells under three different light intensities and re-suspended them in nitrate-free medium at an optical density of approximately 3.0 (OD_730_). Figure 4 illustrates that indeed higher light fluxes can be used to drive faster production of organic acids. We note that the cultures used in this experiment were at a higher optical density than shown for Figs 1 and 3, explaining the higher yields of organic acids. There is also a longer period of linear production of acids (longer living photo-catalyst) in Fig. 4 that may be because of diminished photo-degradation of the cells due to shading effects from high cell densities. The improved productivity was realized only with cultures adapted to the higher light conditions prior to nitrogen starvation (Fig. S1). This observation suggests that once in the photo-catalytic state, the cells are not able to increase productivity under higher light intensities.

Notably, the improvements in production of pyruvate are higher than for alpha-ketoglutarate upon increasing light intensity, as shown in the products ratios (Fig. 4). This suggests that carbon partition between the two products is adaptable to changes in environmental conditions. One possible mechanism is that activity of enzymes beyond pyruvate in the citric acid cycle become limiting at higher light fluxes. Alternatively, other factors such as the ATP/NADPH ratio could vary between different light intensities, re-directing carbon fluxes towards pathways that require more ATP or NADPH (Pattanayak *et al*., 2014). Further research is needed to understand the regulation of carbon partition in this system, which could lead to strategies to produce more of one or the other organic acid.

### Conclusions

We have extended the initial discovery and demonstrated some key features for enhancing photo-catalytic production of organic acids in nitrogen starved cultures of S6803 Δ*glgC*. We have shown that cultures can transition from growth state to photo-catalytic state when inoculated in medium with limited concentrations of nitrate, that the photo-catalytic state can be reversed by re-addition of nitrogen nutrient and that light levels can be increased to drive faster production of organic acids when presumably limiting, accompanied by changes in the ratio of alpha-ketoglutarate/pyruvate. These features suggest that improved understanding of photo-catalytic state and regulation of carbon partition may be useful to commercial applications.

## Experimental procedures

### Cell culture

Cultures of S6803 Δ*glgC* were grown in modified BG-11 medium comprised of 1X standard BG11 freshwater nutrients (except for nitrogen source) and two additions: 20 mM sodium phosphate (pH 7.1) and 5 μg ml^−1^ gentamicin. The media were typically prepared without NaNO_3_, which was added separately to the indicated final concentrations as needed following preculture biomass accumulation in complete medium (17.6 mM NaNO_3_), harvesting by centrifugation (4000 × g) and washing with nitrogen free BG-11. For all experiments, cultures were maintained in 250 ml Erlenmeyer flasks in growth chambers in the presence of 5% CO_2_ enriched air atmosphere. Continuous (24 h) illumination was provided by cool white fluorescent bulbs with a light flux of ∼50 μE* m^−2^s^−1^ at the surface of the flasks or by cool white LEDs at light intensities as high as 300 μE* m^−2^s^−1^ at the surface of the flasks.

### Determination of organic acids

Aliquots (1 ml) of culture were harvested and centrifuged at 13 000 × g. Supernatants were filtered through 0.20 micron filters prior to injection (100 μl) in an Agilent Technology 1200 Series HPLC fitted with Bio-Rad Aminex HPX-87H column, and eluting with 5 mM sulfuric acid at a flow rate of 0.6 ml min^−1^ and detecting with UV detector at 210 nm. Peak retention times and signal integrals were compared against known standard solutions prepared in modified BG11 media with addition of purchased standards of sodium alpha-ketoglutarate and sodium pyruvate (Sigma-Aldrich, USA). No peaks were observed by high-performance liquid chromatography other than from alpha-ketoglutarate and pyruvate that were attributable to biologically produced compounds. All organic acid measurements were determined as the average of at least three independently grown cultures from identical conditions with error bars in figures representing ± 1 standard deviation.

### Optical density and whole-cell spectra

Culture aliquots (1 ml) were measured in a Beckman Coulter DU 800 Spectrophotometer for whole-cell absorbance wavelength scans or scattering at 730 nm. All optical density measurements were determined as the average of at least three independently grown cultures from identical conditions with error bars in figures representing ± 1 standard deviation.

## Conflict of interest

D. Carrieri and T. Paddock are now employed at Matrix Genetics, LLC, a company that engineers cyanobacteria strains of commercial interest.

## Supporting Information

**Fig. S1.** Pyruvate and alpha-ketoglutarate production of ΔglgC cells under varying illumination following growth in lowest indicated light flux (60 μE m^−2^ s^−1^). Cultures were first grown to mid-log phase under 60 μE m^−2^ s^−1^ light and re-suspended in nitrate-free medium at an optical density (OD730) equal to 0.65 and placed under illumination at the light fluxes indicated in units of μE m^−2^ s^−1^ (60, 130 or 300 μE m^−2^ s^−1^).
