# Supplementary material for: Enhancing photo-catalytic production of organic acids in the cyanobacterium Synechocystis sp. PCC 6803 ΔglgC, a strain incapable of glycogen storage
Source: Microb Biotechnol. 2015 Jan 23;8(2):275–80. doi: 10.1111/1751-7915.12243 (PMC4353341; doi:10.1111/1751-7915.12243)
Supplement: Supplementary file 1 [file mbt20008-0275-sd1.doc]

Supplemental Data for:

**Enhancing photo-catalytic production of organic acids in the cyanobacterium *Synechocystis* sp. PCC 6803 ∆*glgC,* astrain incapable of glycogen storage**

Damian Carrieri^+^, Charlie Broadbent, David Carruth, Troy Paddock^+^, Justin Ungerer, Pin-Ching Maness, Maria Ghirardi, Jianping Yu*

National Renewable Energy Laboratory, 15013 Denver West Parkway, Golden, CO 80401, USA

*Correspondence to: [Jianping.Yu@nrel.gov](mailto:Jianping.Yu@nrel.gov); Phone 303-384-6252; Fax 303-384-7836.

^+^Now at Matrix Genetics, LLC, 1600 Fairview Ave East, Suite 300, Seattle, WA 98102, USA


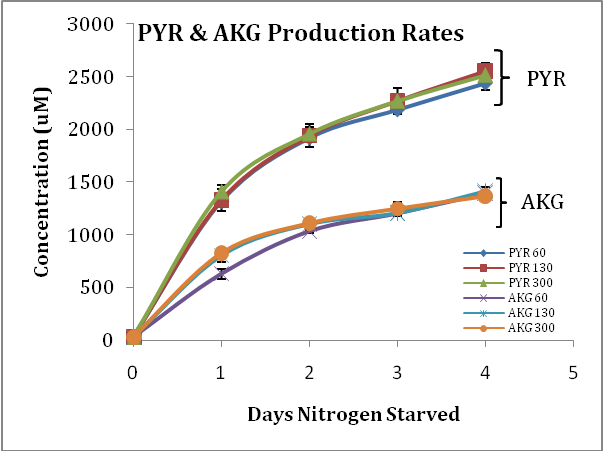


***Figure S1:* PYR & AKG production of Δ*glgC* cellsunder varying illumination following growth in lowest indicated light flux (60 µEm^-2^s^-1^)**. Cultures were first grown to mid-log phase under 60 µEm^-2^s^-1^ light and resuspended in nitrate free medium at an optical density (OD730) equal to 0.65 and placed under illumination at the light fluxes indicated in units of µEm^-2^s^-1^ (60, 130, or 300 µEm^-2^s^-1^).
